# Risk factors and biofilm formation analyses of hospital-acquired infection of *Candida pelliculosa* in a neonatal intensive care unit

**DOI:** 10.1186/s12879-021-06295-1

**Published:** 2021-06-29

**Authors:** Zhijie Zhang, Yu Cao, Yanjian Li, Xufang Chen, Chen Ding, Yong Liu

**Affiliations:** 1 Department of Laboratory Medicine of Shengjing Hospital of China Medical University, 36 Sanhao Street, Heping District, Shenyang, China; 2 College of Life and Health Sciences, Northeastern University, 195, Chuangxin Road, Hunnan District, Shenyang, China

**Keywords:** *Candida pelliculosa*, Fungemia, Antifungal drugs, Infection control, Biofilm

## Abstract

**Background:**

*Candida pelliculosa* is an ecological fungal species that can cause infections in immunocompromised individuals. Numerous studies globally have shown that *C. pelliculosa* infects neonates. An outbreak recently occurred in our neonatal intensive care unit; therefore, we aimed to evaluate the risk factors in this hospital-acquired fungal infection.

**Methods:**

We performed a case-control study, analysing the potential risk factors for neonatal infections of *C. pelliculosa* so that infection prevention and control could be implemented in our units. Isolated strains were tested for drug resistance and biofilm formation, important factors for fungal transmission that give rise to hospital-acquired infections.

**Results:**

The use of three or more broad-spectrum antimicrobials or long hospital stays were associated with higher likelihoods of infection with *C. pelliculosa*. The fungus was not identified on the hands of healthcare workers or in the environment. All fungal isolates were susceptible to anti-fungal medications, and after anti-fungal treatment, all infected patients recovered. Strict infection prevention and control procedures efficiently suppressed infection transmission. Intact adhesin-encoding genes, shown by genome analysis, indicated possible routes for fungal transmission.

**Conclusions:**

The use of three or more broad-spectrum antimicrobials or a lengthy hospital stay is theoretically associated with the risk of infection with *C. pelliculosa*. Strains that we isolated are susceptible to anti-fungal medications, and these were eliminated by treating all patients with an antifungal. Transmission is likely via adhesion to the cell surface and biofilm formation.

**Supplementary Information:**

The online version contains supplementary material available at 10.1186/s12879-021-06295-1.

## Background

*Candida pelliculosa*, also known as *Pichia anomala* or *Hansenula anomala*, is an environmental fungal species frequently isolated from soil, plants, fruits, or organic compounds [1–3]. It is considered a rare fungal pathogen compared with other fungal species such as *Candida albicans* and *Candida parapsilosis*. Clinical analyses reveal that infants, children, and individuals with compromised immune systems, including those from hematologic units, surgical ICUs, and neonatal intensive care units, are more susceptible to bloodstream infections of *C. pelliculosa* that often lead to high morbidity and mortality [4–8]. A recent study found a 41.2% mortality rate in a pediatric ICU [9].

Since the first identified case of infection in the 1950s [8], few outbreaks of fungemia from *C. pelliculosa* have been reported worldwide [10]. To adhere to and transmit between patients, *Candida* species have evolved into sophisticated machineries. For instance, *C. albicans* is capable of forming a biofilm structure by adhering to solid non-biological surfaces such as medical devices. *C. parapsilosis* is frequently found on the hands of healthcare workers. Studies have extensively shown that the expression of adhesion proteins, along with morphological transitions, govern the colonization of *Candida* species [11–17]. The biofilm formed by *C. albicans* has been shown to occur in several stages, including adherence to surfaces followed by hyphal structure formation and the production of an extracellular polysaccharide matrix layer. To initiate the adherence step, *C. albicans* ubiquitously expresses an adhesion molecule known as agglutinin-like sequence 3 (Als3) on the cell surface; this plays an essential role in biofilm formation. The biofilm structure plays crucial roles in fungal adhesion and drug resistance. One study demonstrated that the *C. pelliculosa* biofilm consists primarily of yeast-forming cells [18]; however, very little is known about the molecular nature of its biofilm formation process.

In this work, we report our investigation of an epidemiological outbreak of 21 cases of neonatal fungemia caused by *C. pelliculosa* in the neonatal intensive care unit of a tertiary hospital in northeast China. We systematically analyzed risk factors for *C. pelliculosa* infection, and we phenotypically characterized isolates recovered from the bloodstream. We further analyzed the presence of important genes encoding adhesion proteins, showing that *C. pelliculosa* is capable of forming a biofilm structure on medical devices.

## Materials and methods

### Hospital

Shengjing Hospital of China Medical University is a 6700-bed teaching hospital located in Shenyang in northeast China. It has three neonatal wards in which 140 beds are priority-managed, and tertiary care for patients younger than 28 days of age is delivered. They are open wards with high-frequency doctor and nurse rotations and constant interactions between members of the medical staff. From October 21 to December 142,017, *C. pelliculosa* strains were isolated from blood sampled from 21 newborns on the first and second neonatal wards.

### Clinical characteristics and risk factor analysis

Using these infected newborns, a case-control study was performed to identify the risk factors for *C. pelliculosa* fungemia, enrolling all neonates without fungemia who were born from November 3 to 262,017 as controls. Information was collected from both the treatment and control groups. Data included sex, age at onset of infection (same as hospital day at onset of candidemia), ward, birth weight, gestational age, age of the mother, premature-birth-related problems, delivery type, Apgar scores (at 1 and 5 min), glucose at admission, and length of hospital stay. Also noted were whether any of the following applied: peripherally inserted central catheter (PICC), endotracheal tube, tracheostomy, bladder catheter, umbilical catheter, parenteral nutrition (lipid solution), surgical procedure, broad-spectrum antibiotic use (BSAU), previous or concomitant bacteremic infection (PCBI), three or more broad-spectrum antimicrobials, or a nasogastric tube.

The date of candidemia onset, hospital day (time since admission) of candidemia onset, results of a catheter tip culture, antifungal drug used, and patient outcome were also collected for the study group to reflect clinical characteristics. In addition to the growth of fungi in the blood, the diagnosis of fungal infection was established based on the presence of the following findings: temperature instability (hypothermia, hyperthermia), respiratory issues (grunting, intercostal-subcostal retractions, apnea, tachypnea, cyanosis), cardiovascular disorders (bradycardia, tachycardia, poor perfusion, hypotension), neurological problems (hypotonia, lethargy, seizures), gastrointestinal problems (feeding intolerance, abdominal distension), and laboratory findings, which may indicate an infection, including leukopenia (leukocyte count < 5000/mm^3^), leukocytosis (leukocyte count > 22,000/mm^3^), immature to total neutrophil ratio < 0.2, C-reaction protein (> 8 mg/L), and procalcitonin (> 0.5 ng/mL) [19].

### Outbreak investigation and infection prevention and control

To identify the origin of *C. pelliculosa* in our two neonatal departments*,* epidemiological investigations were performed from November 25 to 30, 2017. Environmental samples were obtained from air conditioners, sinks, refrigerators, tables, patient beds, incubators, trolleys, and respiratory care equipment. Samples from the hands of doctors and nurses were obtained at the same time, resulting in 143 swabs collected for fungus-culturing tests. Outbreak investigations were accompanied by strict infection prevention and control in all wards, and these measures were continued after completion of the investigations. Infected patients were isolated, and uninfected patients were given preventive treatments of fluconazole. Environmental disinfection procedures were carried out three times a day using gamma wipes (Clinell Universal Wipes), air purifiers, and oxyacetic acid fumigation. Furthermore, hand hygiene was practiced before and after each patient contact and before donning or doffing personal protection equipment, and sterilization and daily disinfection of personal protection equipment were employed. Only three cases occurred after these measures were established, all in the first ward and in 2018. No cases occurred in 2019.

### Identification and antifungal susceptibility testing of strains

In vitro cell growth and a mass spectrometer (VITEK MS; bioMerieux, Inc., France) were used to identify cultures isolated from blood samples. If *C. pelliculosa* was identified, an antimicrobial susceptibility test was performed using Fungus 3 (bioMerieux, Inc., France). *C. pelliculosa* drug resistance profiles was performed using the guidelines of the Clinical and Laboratory Standards Institute. Fluconazole, voriconazole, itraconazole, amphotericin B, and flucytosine were selected as antifungal drugs. *Candida parapsilosis* ATCC 22019, *Candida krusei* ATCC 6258, and *C. albicans* ATCC90028 were selected as reference strains. Fluconazole susceptibility (susceptible ≤2, resistant > 4 for non-species related breakpoints for *Candida*) was determined using the European Committee on Antimicrobial Susceptibility Testing (EUCAST) breakpoint [20].

### Pathogenicity investigation

#### Hyphae formation ability

The clinical isolates of *C. pelliculosa* and the reference strains *C. albicans* SC5314 and *Cryptococcus neoformans* H99 were grown in either Spider medium (1% nutrient broth, 1% mannitol, and 0.2% K_2_PO_4_) [11, 12], yeast extract peptone dextrose medium (1% yeast extract, 2% peptone, and 2% dextrose), or Dulbecco’s modified Eagle’s medium (DMEM; supplemented with 10% fetal bovine serum) at 37 °C. After 1 h incubation, fungal cell morphology was examined using a Leica DMI3000 B scanning microscope. Cells with filamentous morphologies are hyphal cells, and those with budding or round morphologies are yeast cells [21].

### Biofilm formation ability

Biofilm formation assays were performed as described elsewhere [17, 22]. Fungal cells were grown at 30 °C in an orbital shaker in liquid yeast extract peptone dextrose medium for 16 h. The fungal cells were washed twice with equivalent volumes of phosphate-buffered saline (PBS; 0.15 M NaCl and 0.03 M phosphate [pH 7.2]) and then diluted to 100 μL with 0.02 of Optical Density (*Absorbance = 600 nm*) in Spider medium and placed in each well of a 96-well plate. The plates were placed on a rotor and agitated at 100 rpm and 37 °C for 2 h (initial adhesion phase). The supernatant cells (planktonic and non-adherent cells) were then removed, and the adherent cells were washed three times with 200 μL PBS. The wells were then filled with 200 μL fresh Spider medium and incubated at 37 °C. After incubating for 48 h, the plates were washed three times with 200 μL PBS. Fungal biofilms were stained by adding 100 μL 0.2% aqueous crystal violet, and the plates were then incubated at room temperature for 15 min and de-stained with 100 μL glacial acetic acid. The adherent cells were analyzed by measuring the crystal violet solution spectrophotometrically at 570 nm [23]. For each clinical isolate of *C. pelliculosa*, five biological replicates were performed. For each biological replicate, five technical replicates were performed.

### Als sequence analysis

The biofilm-related proteins (Als proteins) were identified using protein BLAST (https://blast.ncbi.nlm.nih.gov/Blast.cgi?PROGRAM=blastp&PAGE_TYPE=BlastSearch&LINK_LOC=blasthome), applying the *C. albicans* Als3 protein as the query sequence. They were further determined using Clustal Omega (https://www.ebi.ac.uk/Tools/msa/clustalo/) for multiple sequence alignment analyses to the *C. pelliculosa* genome. The homologous region was highlighted using BoxShade (https://embnet.vital-it.ch/software/BOX_form.html), and a phylogenetic tree was constructed as described elsewhere [24].

### Total RNA isolation and qRT-PCR

To determine adhesin gene expression, total RNA samples were isolated from *C. pelliculosa* clinical strains followed by DNase treatment to eliminate genomic DNA. This was verified using RT-PCR. One microgram of total RNA was used for each strain, then cDNA was synthesized using SuperScript™ III First-Strand Synthesis System kit (ThermoFisher) according to manufacturer instructions and as described elsewhere [15]. Quantitative PCR was performed as described elsewhere [15], and the expressions of the adhesin gene was measured using oligo pairs CpAls4F (gttcagttgttgtctcctcagaa) and CpAls4R (ctctggagttgatgggtattgag), and CaAls3F (atcgcctatcatttcttctagtgct) and CaAls3R (gggtatcagaattggattgcga). *ACT1* were measured using oligo pair ActF (tgctgaacgtatgcaaaagg) and ActR (atccacatttgttggaaagt). Relative expression was calculated using the 2^-ΔΔCt^ method. Standard deviations were calculated using three replicates.

### Ethics approval and consent to participate

All information was anonymized before being made available for research. This study was approved by the Shengjing Hospital of China Medical University Ethics Committee (Approval No. 201PS049K). All methods were carried out in accordance with relevant guidelines and regulations. Informed consent was documented from parents and/or legal guardians.

### Statistical analysis

Statistical analysis was performed using the SPSS 22.0 software package. The χ^2^ test or Fisher’s exact probability method was used for comparing count data between groups. Normally distributed data were represented using the mean (± standard deviation; [$\overline{\upchi}\pm \mathrm{S}$]) or the median (interquartile range). Groups were compared using the *t* test or the Wilcoxon rank sum test. Multivariate analyses of the risk factors for fungemia were performed using unconditional logistic regression, finding statistical significance when *P*<0.05.

## Results

### Clinical characteristics of the patients

Twenty-one infected patients experienced fungemia onset from October 21 to December 14, 2017. Ten were from the second neonatal ward (accounting for 67% of the patients infected in that time frame). The hospital day at fungemia onset ranged from 10 to 42 (Table S1). All patients received interventional procedures: either a PICC, endotracheal tube, nasogastric tube, tracheostomy (without tracheal cut), bladder catheter, or umbilical catheter (Table 1). The catheter tip culture tests from 7 patients were positive for *C. pelliculosa* infection (accounting for 33% of the study group), and the results were consistent with the blood culture tests. Eighteen of the 21 neonates (86%) were treated with broad-spectrum antibiotics (piperacillin/tazobactam, cefoperazone/sulbactam, or meropenem) for documented or suspected bacterial infections prior to fungemia diagnoses. A large majority (20/21, 95%) were treated with fluconazole, and the other was treated with voriconazole (Table S1).

**Table 1:** Univariate analyses of factors associated with *C. pelliculosa* fungemia

Factor	Fungemia	No fungemia	χ2/t/Z	***P***
Sex			0.015	0.904
Male	13	81		
Female	8	47		
Age admitted in hospital (day)	1(1,1)	1(1,1)	0.741	0.642
Ward			1.014	0.314
Neonatal ward 1	9	70		
Neonatal ward 2	12	58		
Age of the mother (year)			9.826	**0.007**
≥ 35	13	39		
30–34	7	49		
≤ 29	1	40		
Gestational age (WK)			28.837	**< 0.001**
≥ 36	0	27		
30–35	6	83		
≤ 29	15	18		
PBRP			1.961	0.744
PRM	3	31		
PHSP	7	35		
Gestational diabetes	0	3		
Twins preterm birth	3	11		
Other	8	48		
Type of delivery			0.121	0.728
Cesarean	15	96		
Natural labor	6	32		
Birth weight			35.849	**< 0.001**
ELBW (≤1000 g)	8	4		
VLBW (> 1000 g, ≤1500 g)	11	32		
LBW (> 1500, ≤2500 g)	1	60		
NBW (> 2500)	1	32		
Apgar (1 min)			27.137	**< 0.001**
≥ 7	6	105		
< 7	15	23		
Apgar (5 min)			0.000	1.000
≥ 7	21	125		
< 7	0	3		
Glucose at admitted (mmol/l)	4.076 ± 1.685	3.681 ± 1.175	−1.567	0.119
PICC			71.406	**< 0.001**
No	0	111		
Yes	21	17		
Endotracheal tube			22.252	**< 0.001**
No	0	71		
Yes	21	57		
Nasogastric tube			1.781	0.182
No	0	16		
Yes	21	112		
Parenteral nutrition (lipid solution)			8.667	**0.003**
No	0	39		
Yes	21	89		
PCBI			1.454	0.228
No	20	105		
Yes	1	23		
BSAU			17.421	**< 0.001**
No	0	62		
Yes	21	66		
TBSA			4.582	**0.032**
No	15	116		
Yes	6	12		
Surgical procedure			0.000	1.000
No	20	121		
Yes	1	7		
Time hospitalization	59.790 ± 21.423	25.190 ± 16.185	−10.146	**< 0.001**

*PBRP* premature birth related problems, *PRM* Premature rupture of membranes, *PHSP* pregnancy-induced hypertension syndrome or preeclampsia, *ELBW* extremely low birth weight, *VLBW* very low birth weight, *LBW* low birth weight, *NBW* normal birth weight, *PICC* peripherally inserted central catheter, *PCBI* previous or concomitant bacteremic infection, *BSAU* broad-spectrum antibiotics use before fungemia, *TBSA* three or more broad-spectrum antimicrobials

In 76% (16/21) of the study group, white blood counts were significantly below the reference range, and in 14% (3/21), they were above the reference range. Similarly, in 71% (15/21), values for C-reactive protein were above normal levels. Finally, 8 of the 9 patients (89%) tested for procalcitonin showed abnormally high values (greater than 0.5 ng/mL; Table S1).

### Risk factor analysis

To analyze potential risk factors for *C. pelliculosa* infection and transmission in our neonatal wards, patient characteristics were compared between the study and control groups. As shown in Table 1, univariate analysis demonstrated that 10 factors were significant: age of the mother, gestational age, birth weight, 1-min Apgar score, use of a PICC, use of an endotracheal tube, use of parenteral nutrition (lipid solution), use of a broad-spectrum antibiotic, use of three or more broad-spectrum antimicrobials, and hospitalization time (Table 1). Further analysis using multivariate analysis revealed that only three or more broad-spectrum antimicrobials and hospitalization time were statistically significant (*P* values = 0.024 and *P* = 0.023, respectively; Table 2).

**Table 2:** Multivariate analyses of factors associated with *C. pelliculosa* fungemia

Factor	OR	95%CI	*P*
Age of the mother (year)			
≥ 35	1.000		
30–34	0.842	0.084 ~ 8.440	0.884
≤ 29	1.610	0.022 ~ 118.431	0.828
Gestational age (WK)			
≥ 36	1.000		
30–35	1166.210	0.000 ~ +∞	0.999
≤ 29	262.736	0.000 ~ +∞	1.000
Birth weight			
ELBW(≤1000 g)	1.000		
VLBW (> 1000 g,≤1500 g)	4.232	0.222 ~ 80.831	0.338
LBW (> 1500, ≤2500 g)	0.681	0.012 ~ 38.272	0.852
NBW(> 2500)	2,141,908.204	0.000 ~ +∞	0.998
Apgar (1 min)			
≥ 7	1.000		
< 7	2.831	0.297 ~ 26.981	0.366
PICC			
No	1.000		
Yes	2.319E+ 10	0.000 ~ +∞	0.997
Endotracheal tube			
No	1.000		
Yes	60,507,419.70	0.000 ~ +∞	0.997
Parenteral nutrition (lipid solution)			
No	1.000		
Yes	204.134	0.000 ~ +∞	1.000
BSAU			
No	1.000		
Yes	0.835	0.000 ~ +∞	1.000
TBSA			
No	1.000		
Yes	0.043	0.003 ~ 0.656	**0.024**
Time hospitalization	1.138	1.018 ~ 1.272	**0.023**

*ELBW* Extremely low birth weight, *VLBW* Very low birth weight, *LBW* Low birth weight, *NBW* Normal birth weight, *PICC* Peripherally inserted central catheter, *BSAU* Broad spectrum antibiotics use before fungemia, *TBSA* Three or more broad-spectrum antimicrobials

### Outbreak investigation

No *C. pelliculosa* was detected in 143 swab samples from the environment, doctors, and nurses. The outbreak investigation failed to produce significant results due to the open ward settings and the high-frequency rotation of doctors and nurses as well as regular contact between medical personnel in both wards.

### Antifungal susceptibility testing and clinical therapy

Antifungal susceptibility analyses showed that across the 21 isolates of *C. pelliculosa,* the minimum inhibitory concentrations of fluconazole, voriconazole, itraconazole, flucytosine, and amphotericin B were 2 to 4 μg/mL, 0.125 to 0.25 μg/mL, 0.125 to 0.25 μg/mL, no more than 4 μg/mL, and no more than 0.5 μg/mL, respectively (Table S1). Sixteen fungal isolates were susceptible to fluconazole (MIC ≤2), while the remaining five isolates were susceptible dose-dependently. Fluconazole was used to treat 20 patients and significantly improved symptoms after treatment for 10 to 21 days. Voriconazole was used to treat the other patient, and recovery occurred after treatment for 3 weeks. Treatment with azole drugs cleared all infections, and all neonates survived.

### Identification of molecular factors for transmission in *C. pelliculosa*

**Fig. 1:**
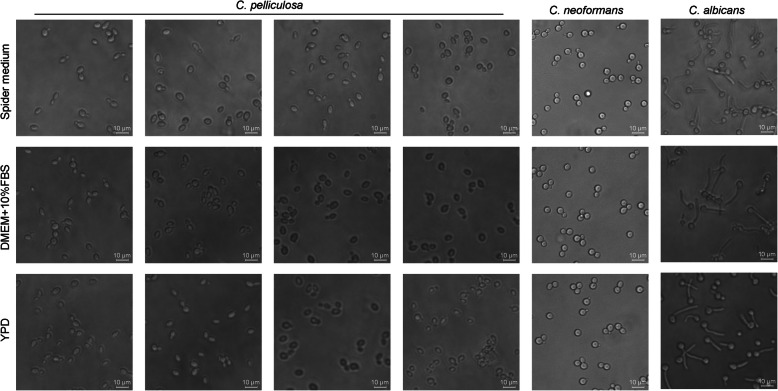
Cell morphological analysis in *C. albicans*, *C. neoformans,* and *C. pelliculosa.* Overnight fungal cell cultures of *C. albicans, C. neoformans,* and *C. pelliculosa* were diluted and subcultured in either yeast extract peptone dextrose medium, Spider medium, or Dulbecco’s modified Eagle’s medium (10% fetal bovine serum). Cultures were incubated at 37 °C for 1 h followed by microscopic analysis. Scale bars represent 10 μm

**Fig. 2:**
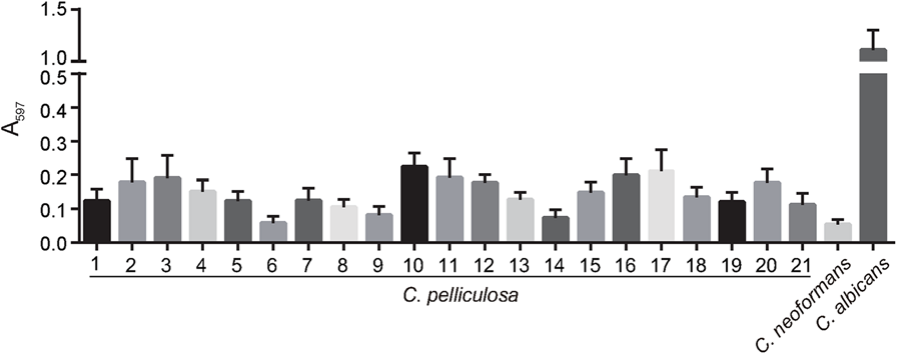
Biofilm assays of *C. albicans, C. neoformans,* and *C. pelliculosa.* Overnight cultures of *C. albicans, C. neoformans,* and *C. pelliculosa* were washed, diluted, and subcultured in Spider medium. The biofilm structures were developed by agitating on a rotor (100 rpm) at 37 °C for 48 h. Biofilm cells were then washed and stained using crystal violet, then quantified at 597 nm The 21 isolates of clinical *C. pelliculosa* and the references strains *C. neoformans* H99 and *C. albicans* SC5314 were tested. Five biological and five technical replicates were performed for each isolate

Numerous studies have extensively demonstrated that biofilm structures facilitate microbial colonization and transmission in the environment. Biofilm formation by *C. pelliculosa* initiates with the adhesion of yeast cells to various surfaces and the subsequent generation of a hyphal structure. Although other fungal species such as *C. parapsilosis* and *C. glabrata* are unable to produce hyphal structures, they can form biofilms. To analyze potential transmission factors during our outbreak, we examined cell morphological switching and biofilm formation in *C. pelliculosa*. A poor biofilm-producing fungal species, *C. neoformans*, was used as a negative control. Differing from the hyphal-forming *C. albicans* SC5314*, C. pelliculosa* and *C. neoformans* H99 showed yeasts and a non-hyphal-cell morphologies, in spite of being cultured in hyphal-inducing media (Spider medium and DMEM; Fig. 1). Biofilm analyses using 96-well plates demonstrated that all *C. pelliculosa* strains were able to produce biofilms, but they were weaker in *C. pelliculosa* compared to *C. albicans* SC5314. The trend was to form 2- to 3-fold more biofilm in 18 isolates (not in isolates 6, 9, or 14) than in *C. neoformans* H99 strain (Fig. 2).

**Fig. 3:**
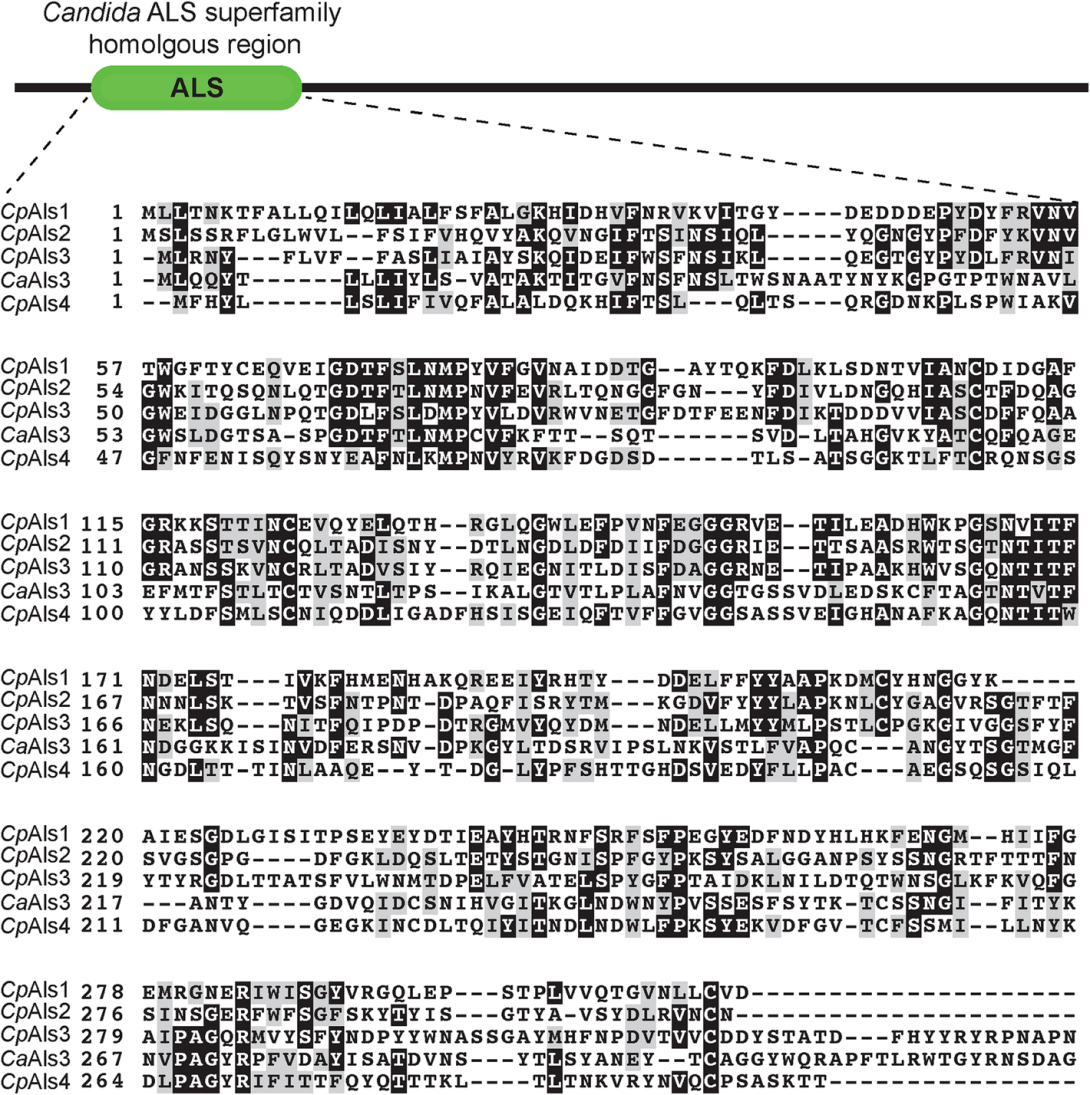
Multiple alignment of Als proteins from *C. albicans* and *C. pelliculosa.* The *C. albicans* Als3 protein is labelled CaAls3, whereas the *C. pelliculosa* Als homologs are labelled CpAls1–4. The National Center for Biotechnology Information accession numbers are: CpAls1, XP_019036962.1; CpAls2, XP_019037149; CpAls3, XP_019038428.1; and CpAls4, XP_019040826

**Fig. 4:**
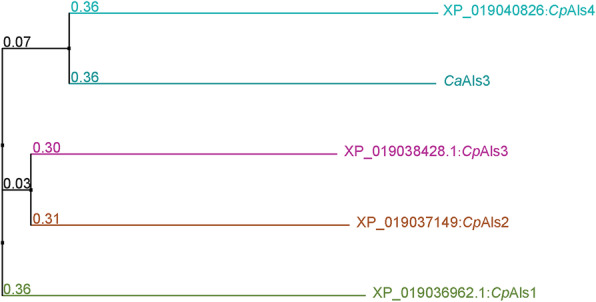
Phylogenic analysis of the Als family in *C. albicans* and *C. pelliculosa.* Numbers indicate the level of bootstrap resampling. *Candida albicans* Als3 protein is labelled *Ca*Als3. Homologs of *C. pelliculosa* Als are labelled with their National Center for Biotechnology Information accession numbers and serial numbers

**Fig. 5:**
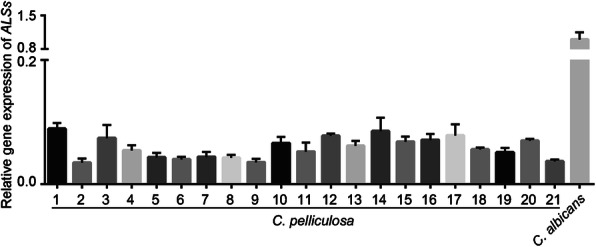
Results of RT-PCR analysis of the expression of *ALS4* in *C. pelliculosa*. The gene expression of *ALS4* in *C. pelliculosa* was confirmed using RT-PCR, isolating mRNA samples from 21 isolates of *C. pelliculosa*. cDNA was synthesized as described in Material and Methods. The endogenous control was *ACT1*. The C_T_ values were calculated using the 2^-ΔΔCt^ methods

The genome of *C. pelliculosa* was searched and analyzed for the presence of homologs to *C. albicans* Als3 (a major biofilm adhesion molecules family). The protein sequence of *C. albicans* Als3 (*CaAls3*) was used to BLAST the *C. pelliculosa* genome, and four proteins were found to share significant Als3 similarity: XP_019040826 (*CpAls4*), XP_019038428.1 (*CpAls3*), XP_019037149 (*CpAls2*), and XP_019036962.1 (*CpAls1;* Fig. 3). Using phylogenic relationship analysis, XP_019040826 (*CpAls4*) was shown to be the closest homolog to *C. albicans* Als3 (31% similarity; Fig. 4). To confirm the expression of Als4 in *C. pelliculosa*, RT-PCR was performed, showing that all isolates express *ALS4* (Fig. 5). The biofilm data and genome analysis demonstrated the presence of functional cell surface adhesion proteins, and the ability to produce biofilm structures contributed to the high rate of transmission of *C. pelliculosa* in our neonatal intensive care unit.

## Discussion

*Candida* species are life-threatening fungal species that cause bloodstream infections worldwide. An opportunistic fungus belonging to the *Candida* clade, *C. pelliculosa* is evolutionarily related to the deadliest fungus, *C. albicans* [4, 6, 25–30]. Recent studies have demonstrated that the infection caused by *C. pelliculosa* is very common in neonates [31, 32].

In this study, we extensively investigated potential risk factors for infection, using univariate analysis to find a significant correlation between 10 individual factors with fungemia, including gestational age, birth weight [31, 32], use of a dwelling central venous catheter [4, 9, 31], use of parenteral nutrition [4, 31], and use of an endotracheal tube [4]. Studies have shown that lower gestational age and lower birth weight are important risk factors for *C. pelliculosa* fungemia [31, 32], and our study supports that. Because neonates with these conditions are often accompanied by impaired or immature immune systems, they are easily invaded by various infectious fungal species [32]. Invasive treatments are considered critical risk factors for *Candida* infections. Studies have shown that use of a dwelling central venous catheter [4, 9, 31], parenteral receipt of complete nutrition [4, 31], and mechanical ventilation [4] present essential risks for *C. pelliculosa* infection. Our data support this: the use of an endotracheal tube or a PICC were significantly correlated with *C. pelliculosa* infection; however, no significant difference was found with the use of a nasogastric tube.

In addition, multivariate analysis found that the use of three or more broad-spectrum antimicrobials or hospitalization time played critical roles in infection acquisition, demonstrating the significance of evaluating these factors for acquired hospitalized infection. Although we were unable to identify the origin of the outbreak in our facility (the open ward environment and frequent contact with individuals), infection was significantly controlled using strict infection prevention and control procedures.

We failed to identify breakpoints in *C. pelliculosa* drug resistance profiles using the guidelines of the Clinical and Laboratory Standards Institute, and this led to ineffective identification of drug sensitivities in this fungus. The breakpoint for *C. pelliculosa* has not been demonstrated by CLSI or EUCAST, so non-species related breakpoints for *Candida* were chosen as a guide (from https://www.eucast.org/fileadmin/src/media/PDFs/EUCAST_files/AFST/Clinical_breakpoints/AFST_BP_v10.0_200204_updated_links_200924_final.xlsx) [20]. All tested strains demonstrated fair sensitivities to the anti-fungal agents tested (MIC < 4), and no drug-resistant isolates were identified. By treating with either fluconazole or voriconazole, all our patients survived. Similar survival rates have been reported: all patients survived the outbreaks in Brazil [8, 31, 33] and Turkey [29]. However, high mortality rates have also been reported, ranging from 16.7 to 42.2% in India [32], Croatia [5], Taiwan [6], South Korea [4], and the 2005 outbreak in Brazil [9]. The fluconazole-tolerant concentration reported in Taiwan and South Korea was approximately 2 μg/mL, consistent with our data (2–4 μg/mL). We therefore speculate that the reported high mortality rates could have been attributable to fungal pathogenicity, care protocols, or patient immunity.

Others have demonstrated that fungi, *Candida* species in particular, are capable of expressing adhesion proteins to form strong and sticky biofilm structures which become infection sources for transmission and fungemia [34–36]. *C. albicans* cells can generate hyphal structures upon incubation in hyphal-inducing media, wherein hyphae-specific adhesion proteins, including the major biofilm regulators Als3 and hyphal wall protein 1 are expressed on the hyphal cell wall [11, 12, 37]. These proteins play critical functions in fixing fungal cells on various surfaces such as medical devices and mucosal surfaces. Most importantly, *C. albicans* Als3 is strongly associated with tissue invasion, which leads to candidiasis [37].

Similar to *C. albicans*, *C. pelliculosa* is capable of expressing cell surface adhesin and producing a biofilm structure. Despite the phylogenic analysis of the Als family demonstrating weak support as a result of the limited number of genes characterized, *C. pelliculosa* Als4 indeed showed significant motif conservation in the Als domain. Therefore, the adhesion of *C. pelliculosa* to surfaces is similarly achieved through cell surface adhesins such as the Als3 homologs of *C. albicans*.

In summary, we employed two analyses strategies in this study, univariate and multivariate, to uncover the possible risk factors for hospital-acquired *C. pelliculosis* infection in our neonatal intensive care unit. We were able to successfully identify two essential factors relevant to fungal transmission. However, the research has some limitations. For example, due to the complexity of the environment and the open ward layout settings in our unit, the outbreak investigation failed to yield concrete results. The gene expression of many adhesins in *C. pelliculosa* was discovered through genomic research. As a result, *C. pelliculosa* transmission is most likely similar to that of other *Candida* species. We are presently focusing on a gene disruption method in *C. pelliculosa* to better understand how these adhesive genes manipulate biofilm formation and transmission.

## Conclusion

This study showed the use of three or more broad-spectrum antimicrobial agents, as well as hospitalization time, were critical risk factors in an outbreak of *C. pelliculosa* in our neonatal intensive care unit. Infection prevention and control measures, as well as antifungal treatment, greatly slowed the infection’s spread in the environment and among patients. *C. pelliculosa* has an intact capacity for biofilm formation and adhesive gene expression suggesting potential routes of transfer. More research into the function of adhesive genes is required to better understand of *C. pelliculosa* transmission and develop possible preventive strategies. Our research identified important risk factors for *C. pelliculosa* infection in a neonatal intensive care unit, highlighting the significance of evaluating factors for fungal transmission.

## Supplementary Information

**Additional file 1: Table S1.** Clinical characteristics of patients with *C. pelliculosa* fungemia.

## Data Availability

The datasets used and /or analyzed during the current study are available from the corresponding author on reasonable request.
