# Supplementary material for: Risk factors and biofilm formation analyses of hospital-acquired infection of Candida pelliculosa in a neonatal intensive care unit
Source: BMC Infect Dis. 2021 Jun 29;21:620. doi: 10.1186/s12879-021-06295-1 (PMC8244135; doi:10.1186/s12879-021-06295-1)
Supplement: Supplementary file 1 — Additional file 1: Table S1. Clinical characteristics of patients with C. pelliculosa fungemia. [file 12879_2021_6295_MOESM1_ESM.pdf]

Risk factors and biofilm formation analyses of hospital-acquired infection of *Candida pelliculosa* in a neonatal intensive care unit

Zhijie Zhang[1], Yu Cao[1],Yanjian Li[2], Xufang Chen[1], Chen Ding[2], Yong Liu[1*]

[1]Department of Laboratory Medicine of Shengjing Hospital of China Medical University, 36 Sanhao Street, Heping District, Shenyang, China.

[2]College of Life and Health Sciences, Northeastern University, 195, Chuangxin Road, Hunnan District,Shenyang, China.

*Corresponding author：

Tel: +86-24-96615-72124

E-mail: liuy@sj-hospital.org (Yong Liu)

Table S1 Clinical characteristic of patients with *Candida pelliculosa* fungemia

No	Sex	Onset of candidemia (hospital day)	Ward	BW(g)/GA(WK)	AM	Apgar (1 /5 min)	BSAU	PCBI	catheter tip culture	Inflammatory biomarker				MIC of Antifungal (μg/ml)					Antifungal therapy	outcome
										WBC(×10^9/ml)	PLT(×10^9/ml)	CRP (mg/L)	PCT (ng/ml)	FCA	VRC	5-FC	AMB	ITR		
1	M	Oct 21,2017(21)	2	1234/28	33	7/9	Yes	No	NG	32.70↑	264	10.10↑	—	2	0.25	≤4	≤0.5	0.25	FCA	Sur
2	F	Nov 03,2017(22)	1	1157/28	35	9/10	Yes	No	NG	25.20↑	45↓	79.70↑	65.14↑	2	0.125	≤4	≤0.5	0.25	FCA	Sur
3	M	Nov 04,2017(42)	2	810/27	31	4/8	Yes	No	NG	14.05↓	84↓	94.70↑	—	4	0.25	≤4	≤0.5	0.25	FCA	Sur
4	F	Nov 05,2017(10)	2	1300/33	23	6/9	Yes	No	NG	11.38↓	130	9.86↑	—	2	0.125	≤4	≤0.5	0.25	FCA	Sur
5	F	Nov 05,2017(23)	1	846/28	37	5/9	Yes	No	CP	9.19↓	156	8.83↑	0.59↑	2	0.125	≤4	≤0.5	0.25	FCA	Sur
6	M	Nov 06,2017(18)	2	928/29	36	2/7	No	No	NG	13.83↓	312↑	16.10↑	—	2	0.125	≤4	≤0.5	0.125	FCA	Sur
7	F	Nov 05,2017(12)	2	1208/31	33	7/9	Yes	No	NG	6.46↓	111	7.90	3.10↑	2	0.125	≤4	≤0.5	0.25	FCA	Sur
8	M	Nov 15,2017(42)	2	823/27	38	4/7	Yes	No	CP	15.81	149	2.17	—	2	0.125	≤4	≤0.5	0.25	FCA	Sur
9	M	Nov 15,2017(22)	2	1600/28	38	6/9	No	No	NG	2.14↓	28↓	28.20↑	—	2	0.25	≤4	≤0.5	0.25	FCA	Sur
10	F	Nov 16,2017(13)	2	1170/32	35	7/9	Yes	No	CP	3.20↓	85↓	7.48	—	2	0.125	≤4	≤0.5	0.25	FCA	Sur
11	M	Nov 16,2017(29)	1	3415/35	20	5/9	Yes	No	NG	10.94↓	84↓	14.70↑	0.52↑	2	0.25	≤4	≤0.5	0.25	FCA	Sur
12	F	Nov 17,2017(11)	2	970/29	37	6/8	No	No	NG	11.26↓	202	1.31	—	4	0.25	≤4	≤0.5	0.25	FCA	Sur
13	M	Nov 21,2017(37)	1	735/26	38	2/7	Yes	No	CP	21.30↑	20↓	87.40↑	33.80↑	4	0.25	≤4	≤0.5	0.25	FCA	Sur
14	F	Nov 24,2017(19)	1	1287/29	38	9/9	Yes	Yes (*S.epidermidis*)	CP	15.84	132	10.50↑	1.06↑	4	0.25	≤4	≤0.5	0.25	FCA	Sur
15	M	Nov 24,2017(17)	2	1226/28	40	6/8	Yes	No	CP	3.98↓	60↓	9.50↑	—	4	0.25	≤4	≤0.5	0.25	FCA	Sur
16	F	Nov 26,2017(32)	2	700/26	31	—	Yes	No	NG	4.10↓	136	17.30↑	—	2	0.25	≤4	≤0.5	0.5	FCA	Sur
17	M	Dec 14,2017(18)	1	1045/29	39	6/9	Yes	No	NG	2.78↓	107	13.10↑	0.59↑	2	0.125	≤4	≤0.5	0.25	FCA	Sur

18	M	Dec 14,2017(19)	2	1420/31	30	3/8	Yes	No	NG	6.19↓	159	10.70↑	—	2	0.125	≤4	≤0.5	0.25	FCA	Sur
19	M	Feb 17,2018(33)	1	955/28	30	3/9	Yes	No	NG	7.03↓	381↑	1.07	—	2	0.125	≤4	≤0.5	0.25	FCA	Sur
20	M	Aug 31,2018(22)	1	1214/32	36	8/10	Yes	No	CP	3.58↓	91↓	14.40↑	0.39	2	0.125	≤4	≤0.5	0.25	FCA	Sur
21	M	Sep 28,2018(10)	1	1117/26	31	5/9	Yes	No	NG	10.0↓	103	7.24	0.50↑	2	0.125	≤4	≤0.5	0.25	VRC	Sur

M=male, F=female, BW=birth weight, GA=gestational age (WK), AM=age of mother, BSAU=Broad spectrum antibiotics use before fungemia, PCBI=Previous or concomitant bacteremic infection, NG= no growth, CP=*C. pelliculosa*, WBC=white blood cell, PLT= platelet, CRP=C reaction protein, PCT=procalcitonin ↑increased than reference range↑decreased than reference range, Sur=survival, FCA= fluconazole, VRC= voriconazole, 5-FC=5-fluorocytosine, AMB=amphotericin B, ITR=itraconazole.
